# Design and Implementation of an Intelligent Windowsill System Using Smart Handheld Device and Fuzzy Microcontroller

**DOI:** 10.3390/s17040830

**Published:** 2017-04-11

**Authors:** Jing-Min Wang, Ming-Ta Yang, Po-Lin Chen

**Affiliations:** Department of Electrical Engineering, St. John’s University, No. 499, Sec. 4, Tam King Rd., Tamsui Dist., New Taipei City 25135, Taiwan; mtyang@mail.sju.edu.tw (M.-T.Y.); a87875169@gmail.com (P.-L.C.)

**Keywords:** intelligent windowsill, smart handheld devices, environment sensors, fuzzy microcontroller, Arduino

## Abstract

With the advance of science and technology, people have a desire for convenient and comfortable living. Creating comfortable and healthy indoor environments is a major consideration for designing smart homes. As handheld devices become increasingly powerful and ubiquitous, this paper proposes an innovative use of smart handheld devices (SHD), using MIT App Inventor and fuzzy control, to perform the real-time monitoring and smart control of the designed intelligent windowsill system (IWS) in a smart home. A compact weather station that consists of environment sensors was constructed in the IWS for measuring of indoor illuminance, temperature-humidity, carbon dioxide (CO_2_) concentration and outdoor rain and wind direction. According to the measured environment information, the proposed system can automatically send a command to a fuzzy microcontroller performed by Arduino UNO to fully or partly open the electric curtain and electric window for adapting to climate changes in the indoor and outdoor environment. Moreover, the IWS can automatically close windows for rain splashing on the window. The presented novel control method for the windowsill not only expands the SHD applications, but greatly enhances convenience to users. To validate the feasibility and effectiveness of the IWS, a laboratory prototype was built and confirmed experimentally.

## 1. Introduction

With the development of science and technology and the gradual improvement of human living standards, people have a desire for convenient and comfortable living. The significant advances in sensor-based technology have led to the emergence of the smart home and Internet of Things (IoT), providing convenience, comfort and better quality of life for people [1].

A smart home can be viewed as an environment in which computation and communication technologies are employed for the use and control of household appliances remotely or automatically through IoT, to establish an efficient but comfortable living environment for the resident [2]. Various wireless technologies that can support some form of remote data transfer, sensing and control, such as GSM (Global System for Mobile communications) [3,4], ZigBee [5,6,7], Wi-Fi [8,9], and Bluetooth [10,11], have been provided to embed various levels of intelligence for the smart home. Among the popular wireless connections that are often performed in a smart home, Bluetooth has a high potential in becoming an important technology for the IoT in low power, low cost and small devices. In addition, it is embedded in all current smart handheld devices (SHD) and it can work in the absence of a Wi-Fi connection. Thus, it will indirectly reduce the cost of the smart system. Bluetooth wireless technology may be the best choice in smart home applications [12].

The worldwide population is aging at a fast pace. According to a report released by the United Nations [13], the number of people in the world aged 60 years or over is expected to more than double between 2015 and 2050, reaching nearly 2.1 billion. Promoting quality of life for the elderly and disabled is today an attractive issue. Much work has been reported on the smart home for older adults and disabled people [14,15,16,17,18]. Concerning the user-friendly smart environment, providing an ease-of-use monitoring and control device is crucial.

SHD are increasingly becoming ubiquitous due to their powerful hardware and useful features. According to the eMarketer report [19], there will 4.3 billion smartphone users worldwide in 2016, representing 58.7% of the global population. This information is important because it gives an indication of the number of the SHD users for the smart home developed on these devices. SHD now have mobile sensors that perform activity recognition and detect physical activities with no additional sensing hardware [20]. Various studies have been carried out to build smart environments by means of SHD [21,22,23,24]. Most of the studies concerned real-time monitoring or household appliances controlling.

Energy management system is the integration of technologies and services through home networking for an intelligent living environment. There has been a surge of interest in the home energy management system (HEMS) because the IoT holds promise for making homes smarter. The main purpose of the HEMS is not just to minimize the energy consumption but also to ensure customers’ comfort [25,26]. The HEMS could reduce operational cost of electricity by 23.1% or reduce residential peak demand by 29.6% [27]. Energy management of heating, ventilating and air-conditioning (HVAC) systems is a primary concern in building projects, since HVAC represents the highest percentage of energy consumption in electricity among all building services installations and electric appliances. Much effort has been devoted a HVAC control strategy or algorithm for promoting HEMS [28,29]. The studied HVAC can balance home comfort against energy use. However, few of them have endeavored to include the control of curtains and windows.

People want curtains and windows primarily for daylight and secondarily for the view. Furthermore, curtains and windows have a significant impact on the energy consumption and living environment. The use of curtain and window can decrease the indoor temperature in a hot day, and thus saving energy can be expected. Surprisingly, it was found that in an era of ubiquitous mobile technologies, few papers showed the use of SHD in windowsills including electric curtains and electric windows. This paper proposes an innovative use of SHD to monitor and control the designed intelligent windowsill system (IWS) that provides indoor comfort and healthy environment. For the realization of this system, the IWS equips a compact weather station including a variety of off-the-shelf environment sensors that measures indoor illumination, temperature-humidity, carbon dioxide (CO_2_) concentration, outdoor rain and wind direction. Traditional curtains and windows are manually operated, which is always inconvenient to the elderly and disabled. Although electric curtains have appeared in the market, they cannot adapt to climate changes in the environment.

In this paper an attempt has been made to design and implement a real-time monitoring and control windowsill system for the elderly and disabled. The main contributions of the paper are that a novel control method for the windowsill was proposed and a novel mobile application (app) software was developed to make the system easier to operate and to give the room greater comfort. The control strategy of the work was based on fuzzy and direct control and performed by an Arduino microcontroller. Additionally, the proposed IWS can automatically close windows for splashing rain. A laboratory prototype was built and evaluated in various scenarios. The experimental results showed that the system has a smart control on the electric curtain and electric window.

The remainder of this paper is organized as follows. Section 2 describes the architecture of the proposed IWS. In Section 3, the control strategies considering an intelligent windowsill system is presented. The system operation on SHD is illustrated in Section 4. The testing on the laboratory prototype for experimental verification is provided in Section 5. Final conclusions and an outlook for further work are given in Section 6.

## 2. Proposed Intelligent Windowsill System and Architecture

**Figure 1:**
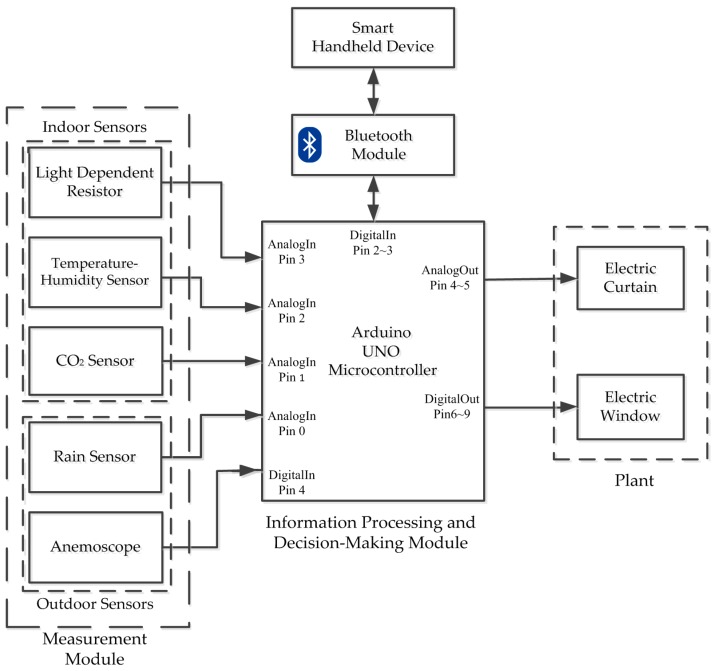
Overall architecture block diagrams of the proposed intelligent windowsill system (IWS).

This section describes the backbone of the system, which comprises several modules. Figure 1 illustrates an overview of the system architecture for the proposed system indicated modules and their interactions. These are “measurement module (compact weather station)” sensing and measuring environment physical quantity and then sending them to Arduino UNO, which is the Arduino’s flagship board; “information processing and decision-making module (Arduino UNO microcontroller)” processing the measured signals and sending an adequate command to windowsill; “Bluetooth module” which provides the wireless communication between SHD and Arduino; “SHD” monitoring the measured information and sending the command to Arduino and the module “plant (windowsill)” which is controlled by Arduino. A brief description of the modules is further explained in the following subsection.

### 2.1. Measurement Module

**Figure 2:**
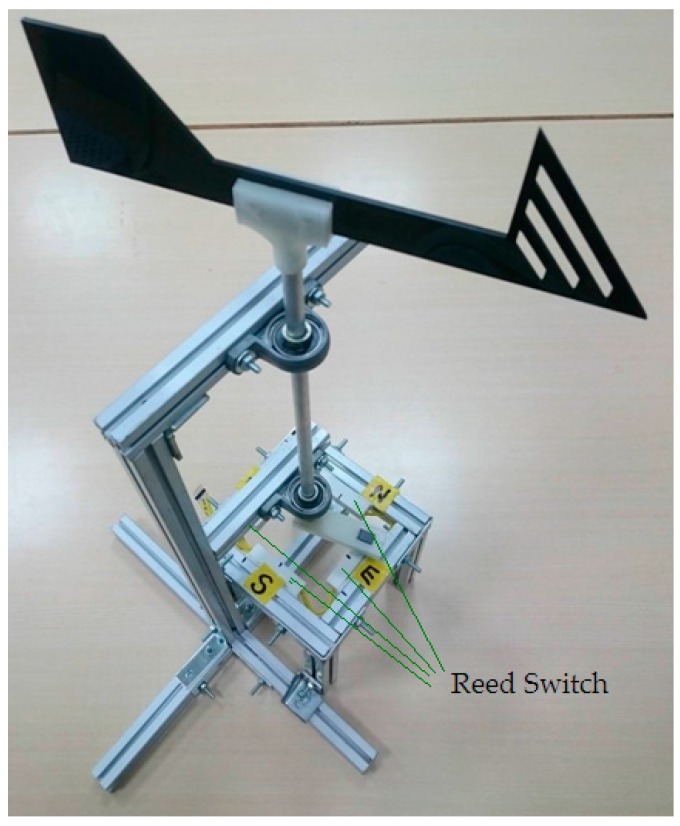
Self-made anemoscope.

The IWS used many types of environment sensors to monitor the real-time status in the home. The measurement module is the key to the IWS and an integration of the indoor and outdoor environment sensors. A light dependent resistor (LDR) or photoresistor is used to measure the indoor illuminance from the daylight contribution and artificial lighting in real-time. The commercial grade Tokenchina’s PGM5537 was used, in which resistance decreases with increasing incident light intensity [30]. The module DHT11 was used for sensing the temperature and relative humidity of air. It measures temperature from 0–50 °C with a precision of ±2 °C and relative humidity ranging from 20%–95% RH with a precision of ±5%. The indoor air quality is mainly influenced by the CO_2_ concentration. The CO_2_ concentration was sensed by using the module MG811, which is a chemical sensor. It works based on the solid electrolyte cell principle. When the sensor is exposed to CO_2_ gas, chemical reactions occur in the cell producing an electromotive force. The CO_2_ detection range of this sensor is 350–10,000 ppm (parts per million) which produces 30–50 mV. In addition, to detect raindrops falling on the electric window, the rain sensor FC-37 was used and mounted on the outside of the window. It runs on a power supply of 5 V. After detecting the raindrop, the sensor board transmits an analog output to the rain sensor driver and then produces a digit output, which will drop from high to low. The digit output will rise from low to high when the raindrop is wiped off or vaporized. To detect the existence and direction of the wind, a self-made anemoscope was built and mounted on the outside of window. The self-made anemoscope shown in Figure 2 is actually much cheaper than those in the market, with no loss in functionality. Four reed switches are respectively placed on the positions of labelled *E* (east), *W* (west), *S* (south) and *N* (north). When the anemoscope is on the windward side, the corresponding reed switch will be activated and a signal will be produced. Once falling raindrops splash against the collected board of the rain sensor and the electric window is on the windward side, a signal will be activated and sent to the Arduino microcontroller for closing the window.

The measured information is gathered and transferred to the information processing and decision-making module, which is an important element for decision-making strategies.

### 2.2. Information Processing and Decision-Making Module

The module is the core of the proposed IWS. It includes two main units: information processing unit and decision-making unit. Considering the environmental conditions provided by the measurement module, the information processing and decision-making module must access the information and provide an adequate command to the windowsill drivers to regulate the plant. The decision-making module provides two ways to control. One way is fuzzy control based on the measured information from indoor sensors for maintaining indoor comfort. The fuzzy control is designed to achieve the requirements based on the designed fuzzy rule base. With the fuzzy control applied, the electric curtain and electric window can maintain their required positions for comfort. Another way is direct control based on the sensed conditions from outdoor sensors for preventing rain from splashing.

Arduinos are used widely by all kinds of makers worldwide. Popularity has been driven by the Arduino’s simplicity of use and the numerous sensors and libraries available to extend the basic capabilities of these controllers [31]. Using such an inexpensive device makes the installation and maintenance of a system easier. In the current set-up, an Arduino is used for interfacing with environment sensors and as a data-logger. Based on the Arduino features, programming in an Arduino UNO containing ATMEGA 328 microcontroller was done to perform the information processing and control the windowsill.

### 2.3. Bluetooth Module

As a communication tool between the control platform and sensor nodes, wireless sensor networks (WSN) have been deployed as a reasonable and low-cost communication technology. In this work, the wireless communication was implemented using the Bluetooth module. Bluetooth is a global wireless communication standard that connects devices together over a certain distance, usually 10 m to 100 m. The work used Bluetooth HC-05 module for the communications between Arduino UNO and SHD.

### 2.4. Smart Handheld Device

A SHD was used to monitor the environment information provided by the measurement module. The user can send a command to the information processing and decision-making module via the Bluetooth module for regulating the electric curtain and electric window appropriately.

**Figure 3:**
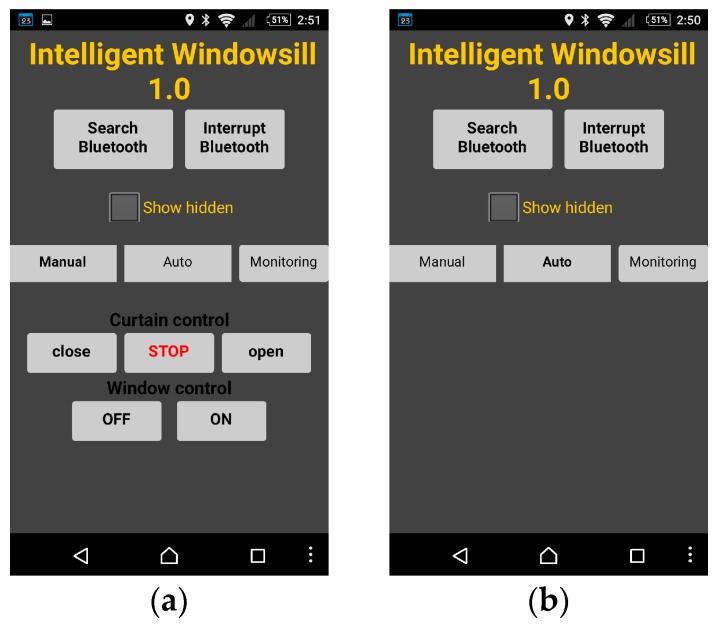
Mobile app: (**a**) The SHD is operated in manual mode; (**b**) The SHD is operated in auto mode.

**Figure 4:**
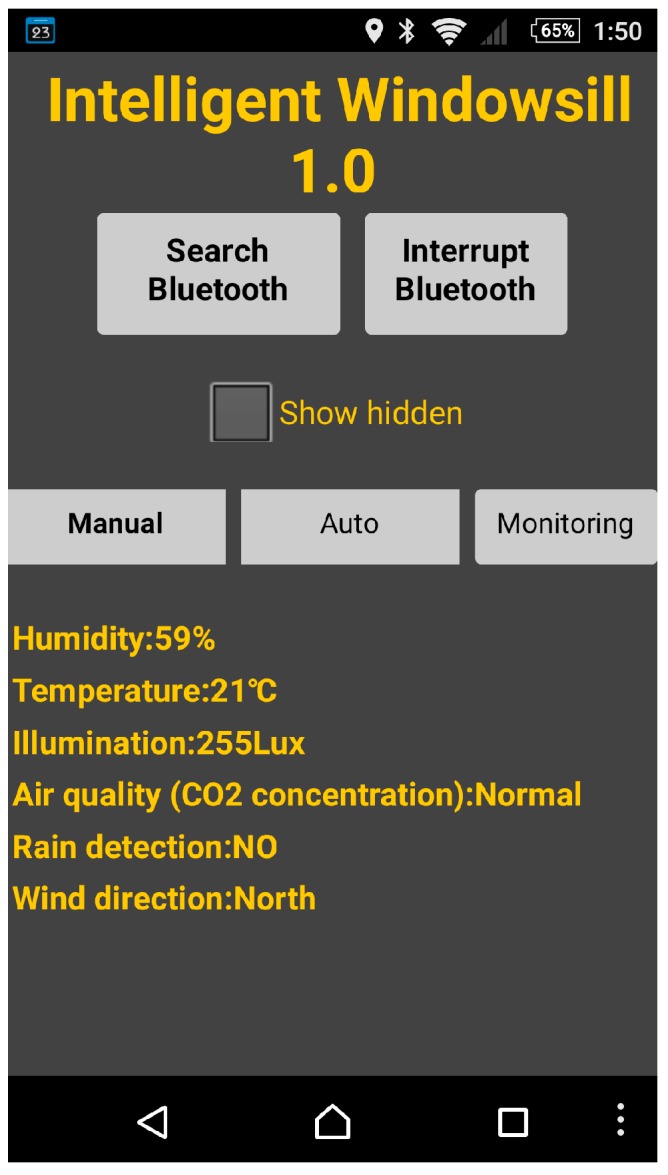
Mobile app shows the monitoring measurement information.

MIT App Inventor is a drag-and-drop visual programming tool for designing and building fully functional mobile apps on the Android based platform. It allows Android apps to be built and programmed with colorful building blocks instead of programming codes. An IWS mobile app was developed using MIT App Inventor 2 for SHD. Three modes of operation can be selected when the SHD is connected to Bluetooth. In Figure 3, two screens of the mobile app are shown. Figure 3a is the default screen that operates in manual mode. The block “show hidden” is a small utility that will display all hidden information on the SHD. The blocks “close” “STOP” and “open” are the selection for controlling the electric curtain, and the blocks “OFF” and “ON” are the selection for controlling the electric window. Figure 3b is the screen of auto mode. Under this mode the windowsill will be automatically controlled based on the design fuzzy rules. In monitoring mode, as shown in Figure 4, the SHD will automatically display the measured information from the measurement module, including humidity, temperature, illumination, air quality, rain detection and wind direction. The shown air quality is normal—that means the range of CO_2_ concentration is from 700 ppm to 2800 ppm in the room. The user can quickly access the measured environment information.

### 2.5. Plant

The plant consists of an electric curtain and electric window. The electric curtain used was the ready-made RAEX M300. Owing to lack of an available electric window, a two-phase six-wire stepper motor with driver module was used to simulate the controlling electric window.

## 3. Fuzzy Control

Fuzzy logic differs from classical logic in that statements are no longer true or false, on or off. In traditional logic, an object takes on a value of either zero or one. However, in fuzzy logic a statement can assume any real value between 0 and 1 representing the degree to which an element belongs to a given set. In contrast to the traditional control theory, fuzzy logic does not need intricate mathematical models to perform, only a practical understanding of the overall system behavior. A fuzzy logic describes a control protocol by means of if–then rules. In engineering systems, it provides a convenient and user-friendly front-end to develop control programs [32,33].

Designing a fuzzy controller is a simple concept that includes the three stages: fuzzification (an input stage), rule evaluation (a processing stage) and defuzzification (an output stage) [32]. The developed fuzzy logic control strategy for the IWS was designed in MATLAB using max–min inference and centroid defuzzification. A comprehensive fuzzy algorithm involving indoor temperature, indoor illuminance and indoor CO_2_ concentration is proposed to evaluate living comfort. The fuzzy inference model is Mamdani with three inputs (indoor temperature, indoor illuminance and indoor CO_2_ concentration) and two outputs (electric curtain and electric window).

**Figure 5:**
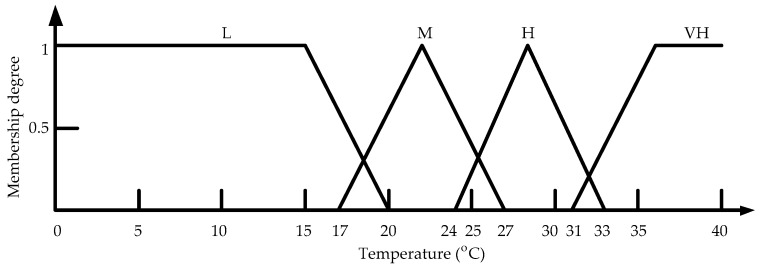
Membership functions for input indoor temperature.

**Figure 6:**
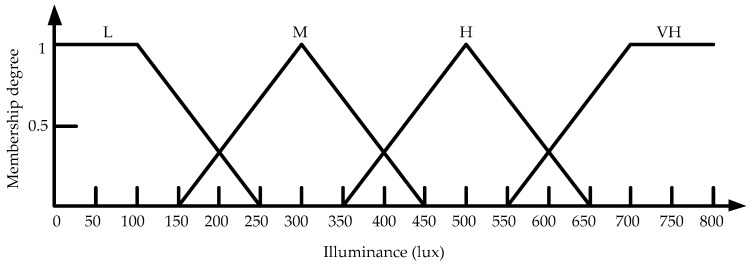
Membership functions for input indoor illuminance.

**Figure 7:**
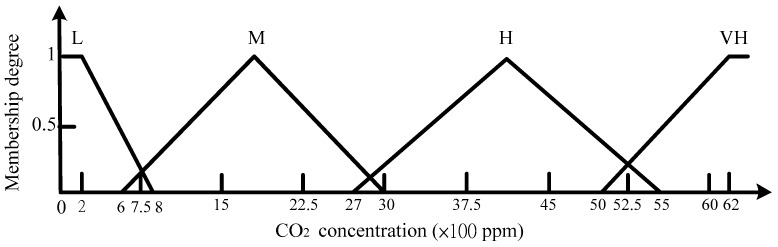
Membership functions for input indoor CO_2_ concentration.

**Figure 8:**
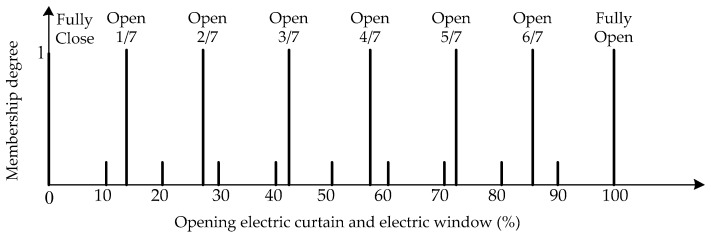
Membership functions for output electric curtain and electric window.

**Figure 9:**
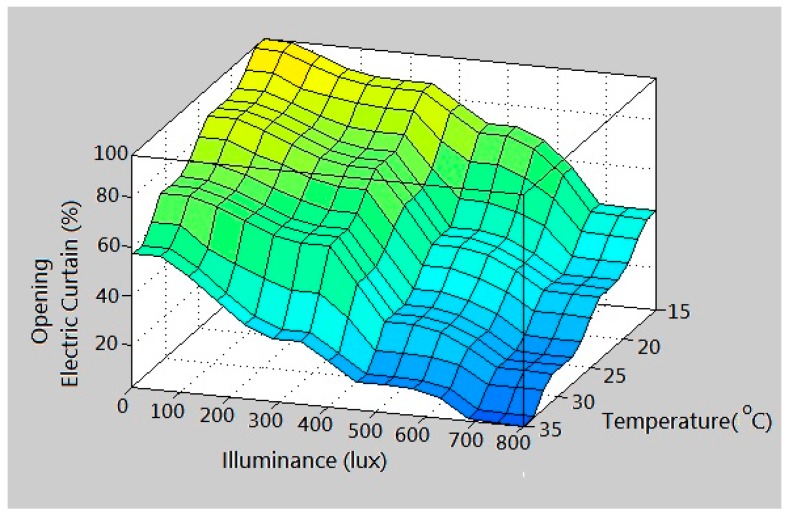
3-D visualization of the inference rules for electric curtain.

**Figure 10:**
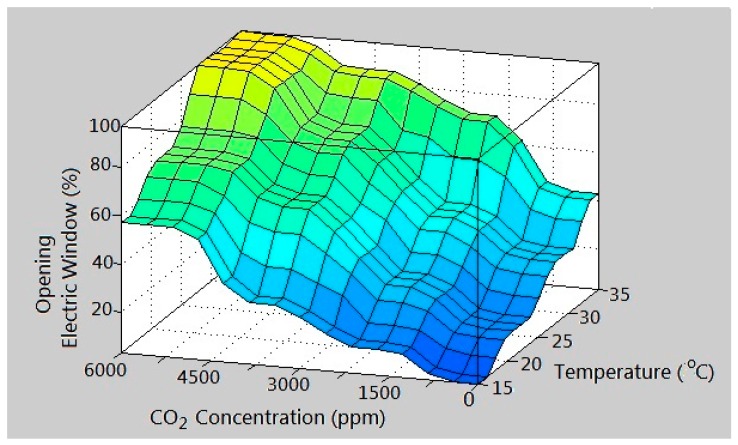
3-D visualization of the inference rules for electric window.

**Figure 11:**
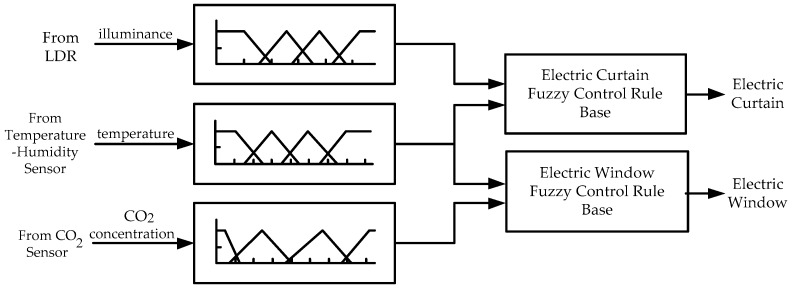
Structure of the fuzzy controller.

The island of Taiwan lies across the Tropic of Cancer. The north of Taiwan is situated in the subtropical region, while the southern part belongs to the tropical climate zone. The studied work was performed in Taiwan. According to the climate in Taiwan, each of the three inputs is defined on the four fuzzy sets {Low (L), Medium (M), High (H), Very High (VH)}. The membership functions of trapezoid and triangular form associated with the fuzzy sets of the input variables are presented in Figure 5, Figure 6 and Figure 7. The membership functions characterize the fuzziness in a fuzzy set. Certainly, the fuzzy sets can be arbitrarily redefined based on the local environment conditions. Through the membership functions, the fuzzy controller evaluates the appropriate outputs for fuzzy rule base that maps inputs to desired outputs. The two outputs of the IWS are described by the eight fuzzy sets {Fully Close, Open 1/7, Open 2/7, Open 3/7, Open 4/7, Open 5/7, Open 6/7, Fully Open}. Figure 8 shows the membership functions for output electric curtain and electric window with all the fuzzy sets. The corresponding fuzzy rule base for the electric curtain with indoor temperature-illuminance is listed in Table 1. Based on the Table 1, the Arduino microcontroller will then make decisions for what action to take. Accordingly, the plot between temperature-illuminance and opening electric curtain is illustrated in Figure 9, which is a 3-D visualization of the inference rules in MATLAB. Similarly, the fuzzy rule base for the electric window with indoor CO_2_ concentration-temperature is listed in Table 2. The corresponding 3-D visualization of the inference rules is also illustrated in Figure 10. The structure of the fuzzy controller is established in Figure 11.

## 4. System Operation on SHD

With regard to the importance of the SHD’s role in human life, a novel Android app was developed to perform the real-time monitoring and smart control for the designed IWS. Concerning cyber security, the first step to protect the IWS from unauthorized users is achieved by the use of passwords. Attempting to operate the IWS for the first time, the user must login with a secret password provided by the Bluetooth HC-05 module manufacturer when the SHD connects to Bluetooth. After passing the system authentication, the IWS can be operated.

**Figure 12:**
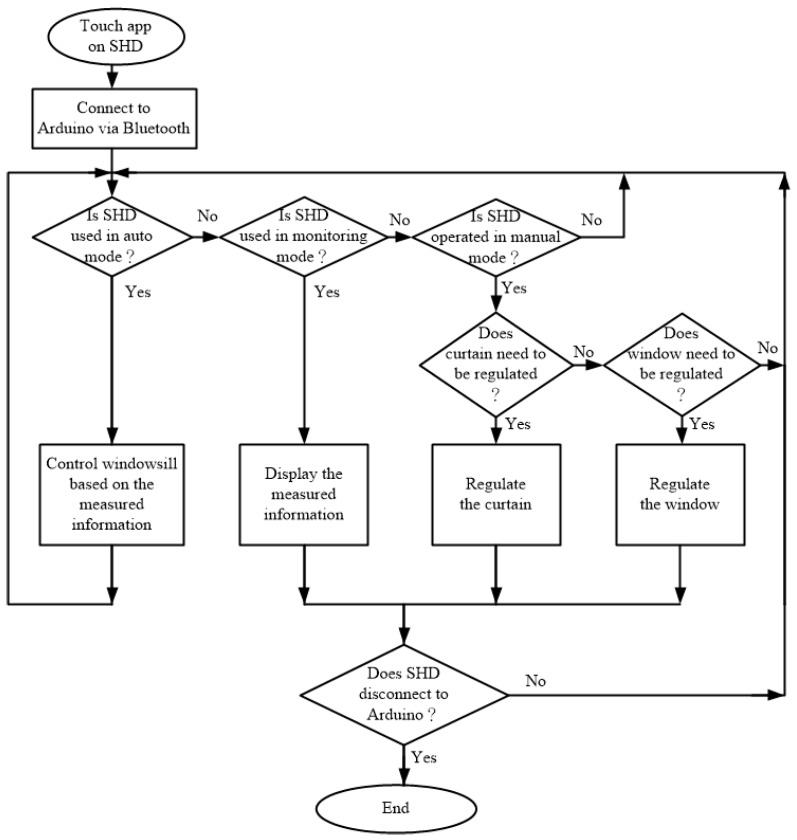
System operation on smart handheld device (SHD) including auto, monitoring and manual modes.

Figure 12 illustrates the system operation on the SHD. The app receives the operations of auto mode, monitoring mode or manual mode from the user. In auto mode operation, the electric curtain and electric window are controlled by the Arduino fuzzy microcontroller based on the measured information and the programming fuzzy rules. In monitoring mode operation, the measured environment information is automatically shown on the SHD as shown in Figure 4. In manual mode operation, the user can arbitrarily control the motions of electric curtain and electric window.

## 5. Experimental Verification

### 5.1. System Implementation

**Figure 13:**
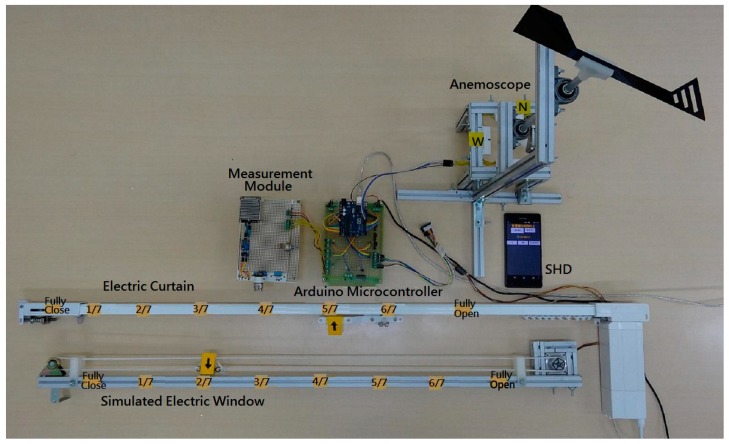
A laboratory prototype of the IWS.

To verify the feasibility and effectiveness of the proposed IWS, a laboratory prototype was built and tested. Figure 13 shows the entire laboratory prototype. The system provides a real-time monitoring for manual and automatic control through the SHD platform. The testing on the laboratory prototype will be evaluated in numerous conditions.

### 5.2. Experiment Results

The goal of this paper was to design an IWS that could monitor the indoor environment information and automatically regulate the electric curtain and electric window for providing indoor comfort and a healthy environment. This subsection is dedicated to verification of the functionality of the IWS, subject to the weather and the rain. The control strategies of the system including fuzzy and direct control will also be examined. Table 3 lists the experiment results for the several scenarios through testing on laboratory prototype. Cases 1–4 are used to evaluate the performance of the fuzzy control. Cases 5–7 are conducted to test the action of the direct control.

**Figure 14:**
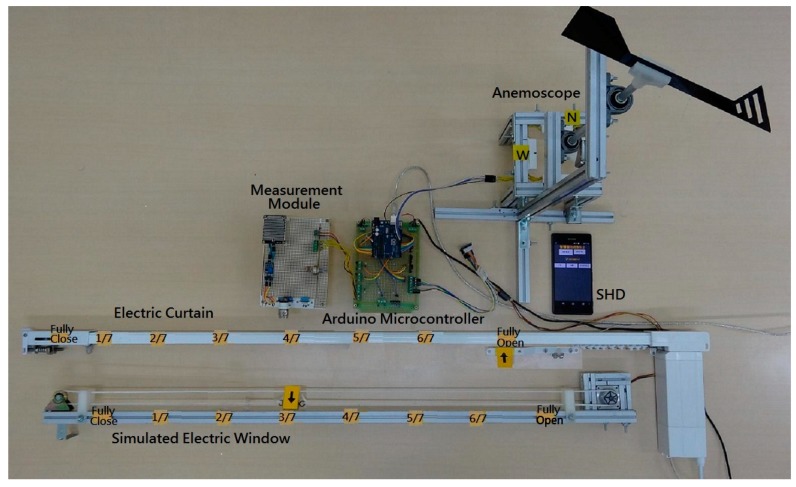
Testing on laboratory prototype for case 1.

Firstly, the operation of auto mode with fuzzy rule bases was made for evaluating the controllability of the Arduino fuzzy microcontroller. In case 1, the indoor illuminance, indoor temperature and indoor CO_2_ concentration are 158 lux, 15 °C and 5200 ppm, respectively. The control status of the IWS is shown in Figure 14. The electric curtain is fully opened and the electric window is opened three-sevenths because of the lower illuminance and lower temperature. Considering the case 2, the indoor illuminance, indoor temperature and indoor CO_2_ concentration are 440 lux, 21 °C and 2200 ppm, respectively. The resulting control actions are that the electric curtain is opened five-sevenths and the electric window is opened two-sevenths. Figure 13 illustrates the control status of the system. Considering the different environmental conditions for the cases 3 and 4, the control actions for the electric curtain and electric window are listed in the third row and fourth low of Table 3, respectively. They comply with the fuzzy rule bases in Table 1 and Table 2.

Observing the results of the cases 1 and 2, increase in indoor illuminance and indoor temperature cause the decrease in opening of the electric curtain. Additionally, as the CO_2_ concentration decreases, the opening electric window decreases. This is consistent with the fuzzy rules base in Table 2. From the cases 3 and 4, the opening electric curtain decreases with increasing the indoor illuminance and indoor temperature. Moreover, opening the electric window increases with increasing the indoor CO_2_ concentration. The experiments show good results with fuzzy control.

**Figure 15:**
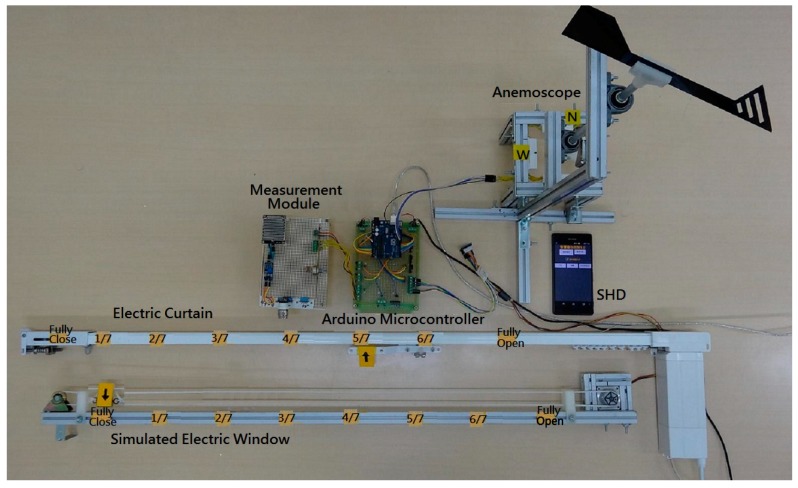
The electric window is fully closed for preventing the rain from splashing in case 7.

Secondly, the prevention of splashing in the rain was verified. Case 5 conducts in falling rain but the window is not on the windward side. In other words, the rain sensor is activated but the anemoscope is not. The statuses of electric curtain and electric window remain unchaged. In contrast to case 5, case 6 has no rain but the window is on the windward side. It has no impact on the electric curtain or electric window. As a result, cases 2, 5 and 6 have the same windowsill status as Figure 13. The final case 7 is where the rain not only beats against the electric window, but the wind blows at the electric window. Both the rain sensor and the anemoscope are activated, and then the Arduino microcontroller sends a command to fully close the electric window and prevent the rain from splashing on the home. Figure 15 illustrates the fully closed electric window. The electric curtain maintains its state, like cases 2, 5 and 6, because the controlling electric curtain is not related to falling rain. The results show the promising outcome with direct control.

## 6. Conclusions

This paper has presented an implementation of IWS utilizing fuzzy control, direct control and Bluetooth wireless technology to develop a novel control method for making the system easier to operate and giving the room greater comfort. To prevent unauthorized users from entering the IWS, password authentication was provided. The remote control and monitoring function by the SHD provided help and assistance, especially to the elderly and disabled. LDR, temperature-humidity sensor and CO_2_ sensor were installed in indoor sites. The rain sensor and anemoscope were placed in outdoor sites. Based on the measured environment information, an Arduino UNO microcontroller was designed to automatically regulate the electric curtain and electric window for responding to climate changes. The developed app available for Android-based SHD allowed remote users to easily browse sensor status and operate the IWS. In addition, integrating the rain sensor and anemoscope, the proposed IWS can fully close the electric window when the rain splashes on the window. A laboratory prototype has been successfully built and tested to verify the effectiveness of the control implementation. Integration of IWS and household appliances such as air conditioner, lighting fixture, air purifier and dehumidifier for creating the smart and energy-saving environment may be future work. The empirical findings may serve as valuable references for further SHD applications and smart homes.

## Figures and Tables

**Table 1:** Fuzzy rule base for electric curtain.

Temperature (°C)	Illuminance (lux)			
	L	M	H	VH
**L**	Fully Open	Open 6/7	Open 5/7	Open 3/7
**M**	Open 6/7	Open 5/7	Open 3/7	Open 2/7
**H**	Open 5/7	Open 4/7	Open 2/7	Open 1/7
**VH**	Open 4/7	Open 2/7	Open 1/7	Fully Close

**Table 2:** Fuzzy rule base for electric window.

Temperature (°C)	CO_2_ Concentration (ppm)			
	L	M	H	VH
**L**	Fully Close	Open 1/7	Open 2/7	Open 4/7
**M**	Open 1/7	Open 2/7	Open 4/7	Open 5/7
**H**	Open 2/7	Open 3/7	Open 5/7	Open 6/7
**VH**	Open 3/7	Open 5/7	Open 6/7	Fully Open

**Table 3:** Testing on laboratory prototype for the evaluation of IWS.

Cases	Illum. (lux)	Temp. (°C)	CO_2_ (ppm)	Falling Rain	Wind Blows at Electric Window	Opening Electric Curtain	Opening Electric Window
1	158	15	5200	No	No	Fully Open	Open 3/7
2	440	21	2200	No	No	Open 5/7	Open 2/7
3	690	24	450	No	No	Open 2/7	Open 1/7
4	976	31	1200	No	No	Fully Closed	Open 5/7
5	440	21	2200	Yes	No	Open 5/7	Open 2/7
6	440	21	2200	No	Yes	Open 5/7	Open 2/7
7	440	21	2200	Yes	Yes	Open 5/7	Fully Closed
